# Age-Related Hearing Loss: Awareness, Perceptions, and Attitudes Among Adults in Saudi Arabia’s Collective Healthcare Context

**DOI:** 10.3390/audiolres16040114

**Published:** 2026-08-06

**Authors:** Eman A. Hajr, Haifa H. Allahem, Aroob M. Alromih, Amal A. Alghtani, Abdullah Z. Alshalan, Faisal N. Almhesen, Abdulrahman A. Hagr

**Affiliations:** 1 Department of Otolaryngology, Imam Mohammad Ibn Saud Islamic University (IMSIU), Riyadh 13317, Saudi Arabia; 2 College of Medicine, Imam Mohammad Ibn Saud Islamic University (IMSIU), Riyadh 13317, Saudi Arabia; 3 King Abdullah Ear Specialist Center (KAESC), King Saud University Medical City, King Saud University, Riyadh 13317, Saudi Arabia

**Keywords:** age-related hearing loss, cochlear implant, Health Belief Model, awareness, perceptions, adults, public health

## Abstract

**Highlights:**

**What are the main findings?**

- Most adults demonstrated good awareness of the signs and consequences of age-related hearing loss, yet significant knowledge gaps persist, including widespread beliefs that hearing loss can be cured or halted, and that cochlear implants benefit children exclusively.
- Despite universal free access to cochlear implantation, awareness gaps rather than structural or financial barriers emerge as the primary obstacle to timely intervention.

**What are the implications of the main findings?**

- In societies with collective healthcare decision-making, family members represent the critical gateway to early identification and treatment of age-related hearing loss; targeted awareness campaigns must therefore reach the family unit, not only older individuals.
- These findings provide foundational data to inform culturally adapted public health strategies and national hearing screening programs in Saudi Arabia and comparable collective healthcare societies worldwide.

**Abstract:**

**Background/Objectives:** Age-related hearing loss (ARHL) is a major and growing public health concern, yet awareness of its nature, consequences, and management options remains inadequately studied in the Middle Eastern context. In Saudi Arabia, cochlear implantation is provided free of charge through the public healthcare system; however, adult uptake remains critically low, suggesting that awareness—rather than access—constitutes the primary barrier to timely intervention. In collective healthcare cultures, family members play a central role in healthcare decision-making for older individuals, making family-level awareness a critical determinant of early identification and treatment. This study aimed to assess public awareness, beliefs, and attitudes toward ARHL and its management options among adults in Saudi Arabia, applying the Health Belief Model (HBM) as a theoretical framework. **Methods:** A cross-sectional descriptive study was conducted using a structured questionnaire adapted from a previously validated survey, with cultural modifications for the Saudi context. The questionnaire was distributed electronically to adults aged 18 years and above across Saudi Arabia. A total of 506 consenting responses were included in the final analysis. Descriptive statistics were used to analyse frequencies, percentages, means, and standard deviations. **Results:** The majority of participants correctly identified hearing loss as a reduction in the ability to hear (84.6%), and 95.3% regarded good hearing as personally important. However, significant knowledge gaps were observed, with 53.8% believing hearing loss can be cured and 50.4% believing it can be halted. Notably, 34.2% believed cochlear implants benefit children exclusively. Doctors and the internet were the primary sources of medical information, yet 39.3% reported no preventive healthcare visits and 23.9% did not seek any information about hearing problems. When faced with a hypothetical hearing loss, 75.3% indicated they would consult an ENT specialist. **Conclusions:** Adults in Saudi Arabia demonstrate good awareness of the signs and consequences of hearing loss but exhibit significant knowledge gaps regarding its clinical course and available management options, most notably the belief that cochlear implantation is exclusively a pediatric intervention. These findings highlight the urgent need for culturally adapted awareness campaigns targeting family members of older adults, and support the development of community-based hearing health promotion strategies in Saudi Arabia and comparable collective healthcare societies.

## 1. Introduction

Hearing loss affects more than 430 million people worldwide and represents one of the leading contributors to years lived with disability globally [1]. With prevalence projected to exceed 700 million by 2050, age-related hearing loss (ARHL) has emerged as a defining public health challenge of ageing societies [2]. Beyond its audiological dimensions, ARHL carries far-reaching consequences across multiple domains of health and wellbeing, including social isolation, emotional distress, increased risk of falls, a higher burden of medical comorbidities, and accelerated cognitive decline [3,4]. Despite these well-documented consequences, ARHL remains systematically underdiagnosed and undertreated—a treatment gap that persists even where clinical services are fully available and accessible.

In Saudi Arabia, this challenge carries particular significance. Data from the General Authority for Statistics Disability Survey indicate that 52.70% of Saudi individuals aged 65 years and above experience some form of difficulty, with hearing difficulties representing the third most prevalent category [5]. According to GASTAT population estimates, individuals aged 65 and above is currently approximately 988,000—a figure projected to grow substantially as life expectancy rises and the kingdom’s predominantly young population ages [6]. Yet the Saudi healthcare landscape reveals a striking paradox: while the state provides free access to advanced rehabilitative care, including cochlear implantation, through Ministry of Health hospitals and affiliated centers, adult uptake remains critically low. Globally, fewer than 2.5% of profoundly deaf individuals have received a cochlear implant, with uptake not exceeding 10% even in high-income countries [7,8]. In Saudi Arabia, a median delay of 17 years has been reported between hearing loss diagnosis and cochlear implantation, with the longest delays occurring prior to specialist referral [9]. This pattern suggests that the primary barrier to timely intervention is not financial or structural, but rather a deficit in awareness at the community level. Understanding where this awareness gap resides requires looking beyond the individual patient. Saudi society is characterized by a collective healthcare culture in which older adults typically reside within extended family households, alongside adult children and grandchildren, rather than independently. These co-residing family members take on responsibility for the older adult’s wellbeing across all domains, including health, and play a decisive role in whether and when help is sought. This observation is supported by national data: the GASTAT Disability Survey noted that difficulties in older adults become apparent not only to the individuals themselves, but to those around them, identifying the family as the primary site of early recognition and the natural gateway to care [5]. If family members lack the knowledge to recognize the signs of ARHL, hold inaccurate beliefs about its nature or treatability, or are unaware of available interventions, the pathway to treatment will remain blocked regardless of the quality of services that exist.

**Figure 1:**
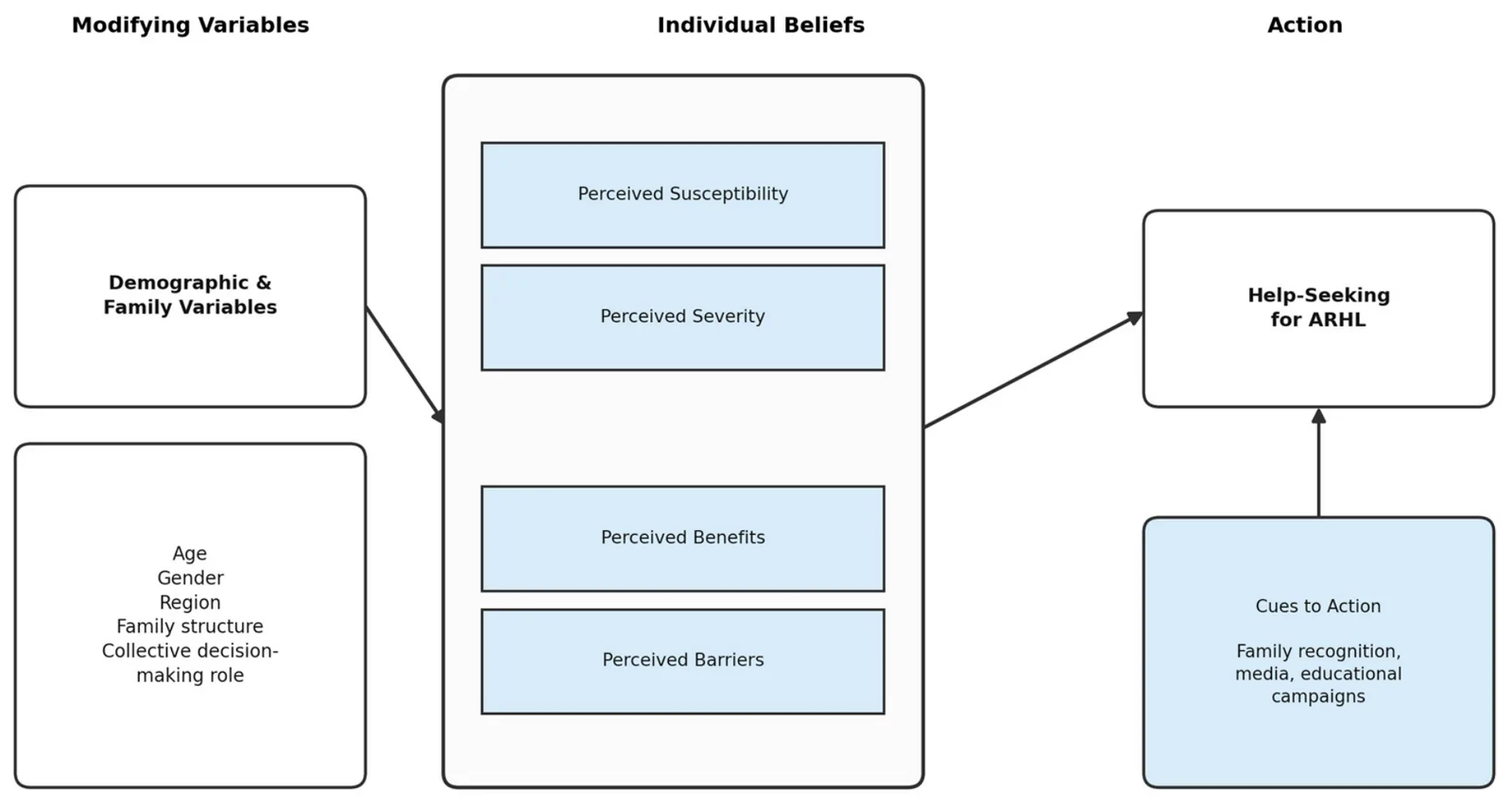
Health Belief Model framework applied at the family level in a collective healthcare context.

The Health Belief Model (HBM) provides a well-established theoretical framework for understanding how personal beliefs and perceptions shape health-related behavior [10]. The model posits that action is more likely when individuals perceive a condition as serious and susceptible to effective intervention, and less likely when perceived barriers outweigh perceived benefits. Figure 1 illustrates this framework as applied at the family level in the present study, adapted from Rosenstock [10] and from a recent qualitative application of the HBM to hearing loss help-seeking among older adults in Singapore [11]. Applying this framework, D’Haese et al. conducted a landmark multinational survey of 500 adults aged 50 to 70 years across five European countries, documenting widespread gaps in knowledge about the nature of hearing loss, its clinical course, and the characteristics of hearing devices [12]. That study established an important evidence based on one confined to European populations and focused on older adults themselves. Comparable data from the Middle East are entirely absent, and no study has applied HBM to assess awareness among the family members who, in collective healthcare cultures, represent the true locus of health decision-making for older individuals.

This study aimed to address that gap by assessing public awareness, beliefs, and attitudes toward ARHL and its management options among adults aged 18 years and above in Saudi Arabia. By targeting the family-level population that surrounds and influences older individuals, and applying the HBM across all adult age groups, the study maps the awareness landscape that ultimately determines whether an older adult in Saudi Arabia reaches timely diagnosis and treatment. The findings provide foundational data to inform culturally adapted educational campaigns, community-based screening strategies, and national health promotion initiatives in the kingdom and across comparable collective healthcare societies worldwide.

## 2. Materials and Methods

### 2.1. Study Design

This study employed a cross-sectional descriptive design to assess public awareness, beliefs, and attitudes toward age-related hearing loss (ARHL) among adults in Saudi Arabia, consistent with the approach adopted in previous European awareness surveys [12].

### 2.2. Questionnaire Development and Adaptation

The survey instrument was adapted from the validated questionnaire developed by D’Haese et al. (2018) [12], with permission granted by the original authors, ensuring that all items were derived from a previously validated, Health Belief Model-based framework rather than newly formulated for this study. The original tool was grounded in the Health Belief Model (HBM) and comprised 13 closed-set questions grouped into three domains: perceived susceptibility to hearing loss and its impact (Questions 3, 4, 5, 10, 12, and 13), perceived barriers to treatment (Questions 1 and 2), and current health-seeking actions (Questions 6, 7, 8, 9, and 11). Several culturally informed modifications were introduced to better reflect healthcare perceptions and decision-making patterns within the Saudi context. All items asked participants to report their own personal awareness, beliefs, and perceptions regarding age-related hearing loss and cochlear implantation, rather than the condition or experience of a specific family member. The rationale for surveying the general adult population, rather than older adults alone, is that family members are typically the first to notice hearing decline and serve as the primary decision-making gateway to care within Saudi Arabia’s collective healthcare culture; the questionnaire therefore captures the personal knowledge and attitudes that inform this gatekeeping role, rather than family members’ assessments of an affected relative. The questionnaire was made available in both Arabic and English, and the Arabic translation underwent forward and backward translation procedures to verify linguistic equivalence. Content validity was further ensured through review by a panel of specialists in otology and audiology. The full questionnaire, including all 13 items and their response options, is provided in Supplementary Material S1.

### 2.3. Pilot Testing

Prior to formal data collection, the questionnaire was piloted on 30 participants drawn from diverse age groups and educational backgrounds. Participant feedback was used to refine item wording, response scales, and overall layout.

### 2.4. Study Population and Sampling

The study targeted adults aged 18 years and above residing in Saudi Arabia, representing a broader age range than the reference European study. This expansion was deliberate and theoretically grounded: in Saudi Arabia, elderly individuals commonly reside within extended family structures, and adult family members play a central role in recognizing health concerns and facilitating healthcare-seeking behavior. Assessing awareness across all adult age groups was therefore considered essential to capture the community-level dynamics that shape early identification and management of ARHL in the Saudi context.

### 2.5. Data Collection

The finalized questionnaire was distributed electronically via social media platforms, university networks, and professional channels to ensure broad demographic representation across regions of Saudi Arabia. Participation was entirely voluntary, and informed electronic consent was obtained from all participants prior to beginning the survey. The minimum required sample size was calculated using Cochran’s formula for an infinite/unknown population, assuming a 95% confidence level (Z = 1.96), a 5% margin of error, and maximum variability (*p* = 0.5): *n* = Z^2^p(1 − p)/e^2^ = 384.16, rounded to 385. Data collection continued until a minimum of 385 responses were achieved, representing the statistically required sample size for a population-level descriptive study. A total of 515 responses were received, of which 506 fully consenting responses were included in the final analysis.

### 2.6. Ethical Approval

This study was reviewed and approved by the Institutional Review Board (IRB) of Imam Mohammad Ibn Saud Islamic University, Riyadh, Saudi Arabia (protocol code 884/2025). The study was conducted in accordance with the Declaration of Helsinki. Informed consent was obtained from all participants involved in the study.

### 2.7. Statistical Analysis

Data was analyzed using the Statistical Package for the Social Sciences (SPSS), version 26 (IBM Corp., Armonk, NY, USA). Frequencies and percentages were reported for categorical variables, and means with standard deviations were reported for continuous variables. As the primary aim of the study was to provide descriptive baseline data, inferential statistical comparisons between subgroups were not the primary focus of the analysis.

## 3. Results

### 3.1. Participant Demographics

A total of 515 responses were received, of which 506 were included in the final analysis following the exclusion of nine non-consenting entries. Demographic characteristics are summarized in Table 1.

### 3.2. Perceived Susceptibility to Hearing Loss and Its Impact (Threat Perception) (Questions 3, 4, 5, 10, 12, and 13)

#### 3.2.1. Beliefs Regarding Cochlear Implantation (Question 3)

**Figure 2:**
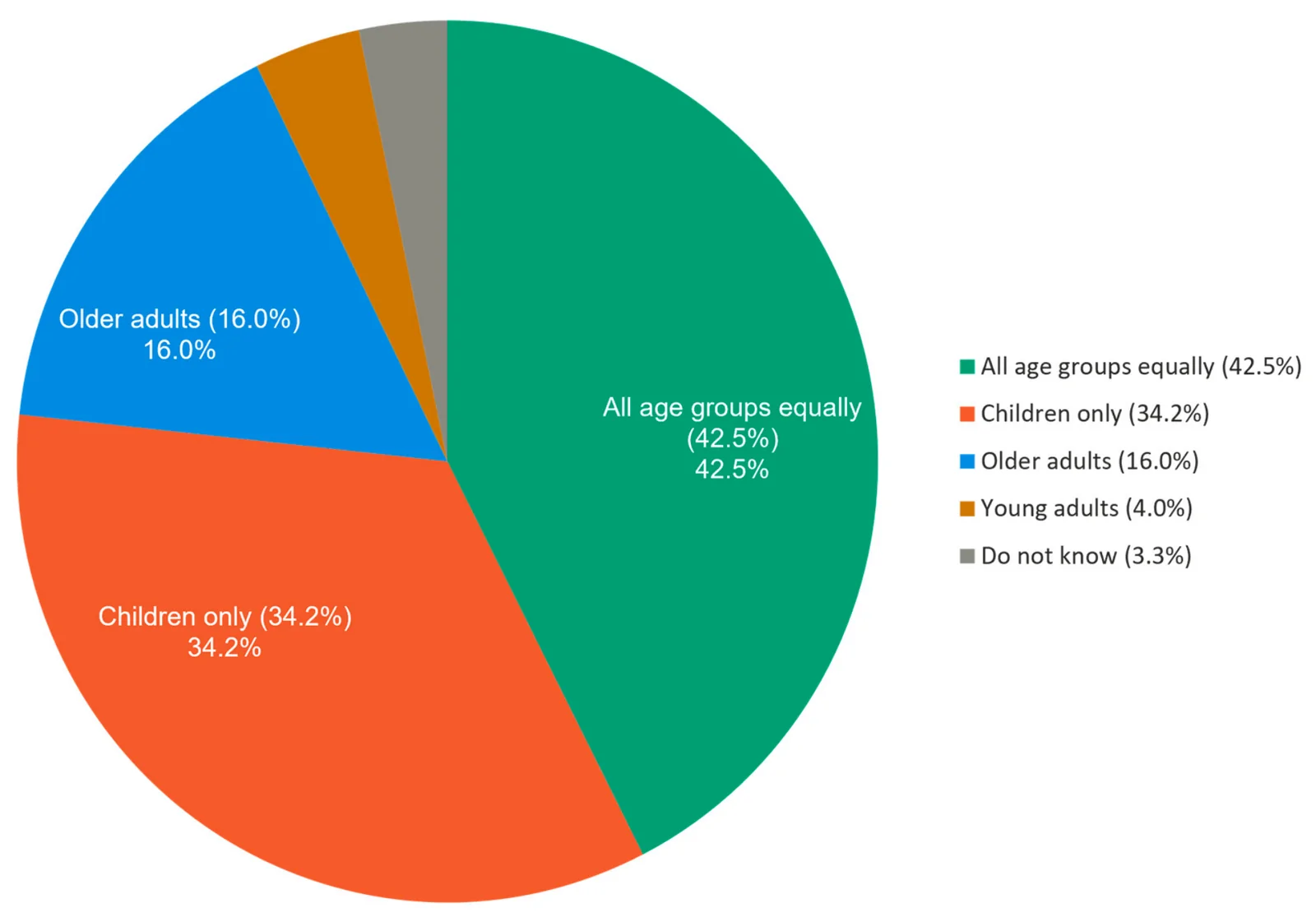
Participants’ beliefs regarding which age group benefits most from cochlear implantation (*n* = 506).

Participants’ beliefs regarding which age group benefits most from cochlear implantation are presented in Figure 2.

#### 3.2.2. Knowledge of Hearing Loss (Questions 4 and 5)

Hearing loss was most commonly identified as a reduction in the ability to hear (*n* = 428, 84.6%). Notable gaps in knowledge regarding the clinical course of age-related hearing loss (ARHL) specifically were observed: more than half believed it can be cured (*n* = 272, 53.8%) or halted (*n* = 255, 50.4%), and 22.1% (*n* = 112) considered it a complete loss of hearing. These beliefs pertain specifically to ARHL, which is generally progressive and irreversible, rather than hearing loss arising from other, potentially reversible, causes. The majority (*n* = 416, 82.2%) recognised a distinction between hearing loss and hearing impairment, while 8.5% (*n* = 43) were uncertain and 5.9% (*n* = 30) believed there was no difference.

#### 3.2.3. Signs and Consequences of Hearing Loss (Questions 10 and 12)

The most commonly recognised sign of hearing loss was increasing the volume of the television or radio (*n* = 430, 85.0%), followed by difficulty localising sounds (*n* = 322, 63.6%), difficulty hearing high-pitched sounds (*n* = 271, 53.6%), tinnitus (*n* = 251, 49.6%), and difficulty using the telephone (*n* = 232, 45.8%). The most frequently selected consequences were restricted communication (*n* = 426, 84.2%), reduced quality of life (*n* = 344, 68.0%), social isolation (*n* = 323, 63.8%), and frustration or emotional distress (*n* = 284, 56.1%).

#### 3.2.4. Personal Importance of Hearing (Question 13)

The vast majority of participants (*n* = 466, 95.3%) indicated that good hearing was very important to them personally.

### 3.3. Actions Taken (Questions 6, 7, 8, 9, and 11)

#### 3.3.1. Sources of Information and Healthcare Behaviour (Questions 6, 7, 8, and 11)

Sources of medical and hearing-related information, precautionary healthcare behaviour, and intended actions in the event of hearing loss are summarised in Table 2.

#### 3.3.2. Reasons for Not Visiting an ENT Specialist (Question 9)

Among participants who did not visit an ENT specialist as a precaution (*n* = 445), the most commonly reported reasons were never having had problems in that area (*n* = 272, 61.1%), never having thought to do so (*n* = 107, 24.0%), and not considering it necessary (*n* = 66, 14.8%).

### 3.4. Barriers to Treatment (Questions 1 and 2)

#### 3.4.1. Perceived Difference Between Hearing Aids and Cochlear Implants (Question 2)

This question was presented to all 506 participants regardless of prior familiarity with either device. When asked whether there is a difference between a hearing aid and a cochlear implant, 76.3% (*n* = 386) answered yes, while 18.4% (*n* = 93) were uncertain and 2.0% (*n* = 10) believed no difference existed. As this was a closed-set, non-verifying question, the relatively high proportion reporting awareness of a difference should be interpreted with some caution, since it does not confirm accurate understanding of what that difference actually is.

#### 3.4.2. Perceptions of Hearing Devices (Question 1)

Perceptions of hearing aids and cochlear implants are presented in Table 3.

## 4. Discussion

This study assessed public awareness, beliefs, and attitudes toward age-related hearing loss and its management among adults in Saudi Arabia, applying the Health Belief Model as a guiding framework. The findings are discussed in relation to the three domains of the HBM: perceived threat, perceived barriers, and current health-seeking actions. These findings are interpreted within a sociocultural context that fundamentally distinguishes this study from previous European research: Saudi Arabia provides cochlear implantation free of charge through the public healthcare system [7,8], elderly individuals overwhelmingly reside within extended family units, and healthcare decisions for older adults are characteristically made collectively. In this context, the family emerges not merely as a social support structure, but as the primary gateway through which older individuals access—or fail to access—timely hearing healthcare.

### 4.1. Perceived Susceptibility to Hearing Loss and Its Impact (Threat Perception)

Most participants (95.3%) regarded good hearing as personally important, consistent with the high valuation of hearing reported across all five European countries in the reference study, where mean scores ranged from 1.2 to 1.4 [12]. This high valuation provides a motivational foundation upon which awareness campaigns can effectively build.

Regarding knowledge of the nature of hearing loss, the majority of participants correctly identified hearing loss as a reduction in the ability to hear (84.6%), broadly consistent with findings across the European cohort, where correct identification ranged from 43% in Sweden to 80% in the United Kingdom [12]. However, significant gaps in knowledge regarding its clinical course were observed: more than half believed that hearing loss can be cured (53.8%) or halted without further deterioration (50.4%)—figures considerably higher than those reported in the European cohort, where equivalent responses ranged from 4% to 9% for curability and 13% to 33% for haltability across five countries. Similarly, 22.1% considered hearing loss to represent a complete loss of hearing ability. These marked differences may reflect the limited availability of public hearing health education in Saudi Arabia and the absence of national awareness campaigns targeting ARHL. Individuals who believe hearing loss is reversible or self-limiting are less likely to seek timely evaluation [13,14] and this gap in knowledge may therefore represent a meaningful driver of delayed care-seeking in the Saudi context.

It is worth noting that ARHL typically begins as a gradual, high-frequency-predominant loss, and not all individuals with ARHL become cochlear implant candidates; candidacy in adults generally requires severe-to-profound hearing loss with poor speech perception despite appropriately fitted hearing aids. As ARHL progresses across the audiogram over time, a meaningful proportion of affected individuals do eventually meet these criteria, underscoring the continued relevance of public awareness across the full course of the disease rather than only at its later stages. A particularly notable finding was that 34.2% of participants believed cochlear implants benefit children exclusively, a culturally specific gap in knowledge not observed at this magnitude in the European reference study. This belief is clinically unfounded: evidence consistently demonstrates that advanced age is not a predictor of poor cochlear implant outcomes, and that older adults achieve hearing and quality-of-life benefits comparable to younger recipients [15,16]. Prevalence data have further shown that hearing loss increases markedly with age, yet provision of cochlear implants in adults over 65 remains disproportionately low relative to younger populations, despite the absence of clinical barriers to implantation in this age group [8]. Addressing this gap in knowledge at the community and family level is therefore a priority for any awareness campaign targeting the Saudi population.

There was strong agreement across participants that the main sign of hearing loss was turning the television or radio volume up high (85.0%), consistent with findings across all five countries in the reference study [12]. The next four most frequently recognized indicators, difficulty localizing sounds (63.6%), difficulty hearing high-pitched sounds (53.6%), tinnitus (49.6%), and difficulty using the telephone (45.8%), were also consistent with the European findings, suggesting that recognition of the cardinal signs of hearing loss is relatively preserved across cultural contexts. Participants demonstrated good awareness of the consequences of hearing loss, with restricted communication (84.2%), reduced quality of life (68.0%), social isolation (63.8%), and frustration (56.1%) being the most frequently reported outcomes.

The combination of high awareness of consequences with significant gaps in knowledge regarding the clinical course of hearing loss suggests a disconnect between understanding the impact of hearing loss and understanding its nature—a pattern that reinforces the need for targeted educational interventions focused specifically on the progressive and irreversible nature of ARHL.

### 4.2. Actions Taken

Consistent with findings from the reference study [12], doctors and the internet were the two most cited sources of both general medical information (45.8% and 35.6%, respectively) and hearing-specific information (36.4% and 32.2%, respectively). However, 23.9% of participants reported not seeking any information about hearing problems—a proportion comparable to the higher end of the European range, where equivalent figures reached 26% in Germany, 31% in France, and 23% in Austria. This suggests that a meaningful proportion of the Saudi public remains disengaged from hearing health information, highlighting the need for proactive outreach strategies.

The finding that 39.3% of participants do not attend any precautionary medical visits in the absence of symptoms is particularly significant, as it reflects a reactive rather than preventive orientation toward healthcare. This pattern aligns with the absence of a national adult hearing screening program in Saudi Arabia, the establishment of which has been advocated as a critical step toward earlier identification and intervention [8,9]. Given that a substantial proportion of the sample was relatively young (46.8% aged 18–30), reduced perceived personal relevance of hearing-related visits likely contributes to this finding, as younger respondents are less likely to have experienced hearing symptoms themselves. However, low precautionary ENT visit rates are not unique to younger samples: D’Haese et al. (2018), despite surveying an older cohort (50–70 years) across five European countries, similarly reported low precautionary ENT visit rates (9–28%) relative to GP or dentist visits [12]. This suggests the pattern reflects a broader gap in awareness of the importance of routine hearing and ear care, rather than being explained by participant age alone. Among those who did attend preventive consultations in the present study, 28.1% reported visiting an ENT specialist, a figure higher than the 9–10% reported in Sweden and the United Kingdom in the reference study, though lower than the 21–28% observed in Germany, France, and Austria.

A striking contrast emerged when participants were asked about their intended course of action in the event of hearing loss: 75.3% indicated they would consult an ENT specialist—a sharp reversal from the 39.3% who reported no precautionary visits. This gap between preventive and reactive healthcare behavior is a central finding of this study. It suggests that the motivation to act exists, but that the trigger—sufficient awareness of personal susceptibility—is absent in the absence of symptoms. This is precisely the mechanism described by the Health Belief Model [10]: action is taken when perceived threat exceeds perceived barriers. In the Saudi context, the threshold for perceived threat has not yet been reached for most adults in the absence of symptoms, even among those who value hearing highly.

### 4.3. Barriers to Treatment

Most participants (76.3%) recognized a difference between hearing aids and cochlear implants, broadly consistent with the European reference study, where recognition of this difference ranged from 40% in France to 72% in the United Kingdom. However, 18.4% remained uncertain, reflecting a persistent gap in knowledge regarding the nature and purpose of cochlear implantation.

Regarding hearing aids, there was good agreement that they must be removed during sleep (62.7% agree), require maintenance (60.2% agree), and can be a hindrance during sports (61.7% agree)—patterns closely mirroring those observed across most European countries. Regarding cochlear implants, participants showed moderate agreement that they are not externally visible (43.6% agree) and are permanently fitted (42.8% agree). Participants tended to disagree with the statement that there is no qualitative difference in hearing sensitivity between hearing aids and cochlear implants (mean = 3.58, 49.2% disagree), suggesting that the majority recognized a functional advantage of cochlear implantation over conventional amplification.

What distinguishes the Saudi context is that the barriers to cochlear implant access are neither financial nor structural. Saudi Arabia provides cochlear implantation free of charge, yet adult uptake remains critically low. This contrasts with settings where cost is a dominant barrier: a qualitative study of older adults in Singapore identified treatment cost as one of the principal deterrents to hearing help-seeking, alongside doubts about hearing aid usefulness [11]. The removal of the cost barrier in Saudi Arabia isolates awareness as the primary remaining obstacle, reinforcing that public education, rather than financial subsidy, is the more urgent priority for this population. Even in countries with government-funded healthcare where financial barriers are minimal, awareness and knowledge gaps have been identified as primary drivers of underutilization [8]. Referral pathway inefficiencies have been documented in both the United Kingdom [17] and the United States [18], where healthcare system factors and referral workflows represent actionable targets for reducing barriers and improving access. It is also worth noting that for patients who are medically unfit for general anesthesia, cochlear implantation under local anesthesia with sedation has been demonstrated to be a safe, effective, and well-tolerated alternative [19,20,21], further reinforcing that clinical suitability is rarely the limiting factor in Saudi Arabia.

### 4.4. The Role of Family Awareness in the Saudi Context

A defining feature of this study—distinguishing it from the European reference study—is its focus on the awareness of family members surrounding older individuals rather than older adults themselves. In Saudi Arabia, older adults typically live within extended family households rather than independently, and healthcare decisions are characteristically made collectively, with co-residing adult children and grandchildren taking responsibility for the older adult’s wellbeing, including hearing health, and playing a central role in initiating help-seeking behavior. National data support this framing: the GASTAT Disability Survey noted that difficulties in older adults become apparent not only to the individuals themselves, but to those around them [5] identifying the family as the primary site of early recognition and the natural gateway to care.

The findings of this study reveal that significant gaps in knowledge—including the belief that cochlear implants are exclusively for children, and that hearing loss can be cured or halted—are prevalent among this family-level population. This has direct practical implications: if family members do not recognize the signs of ARHL, hold inaccurate beliefs about its nature or treatability, or are unaware of available interventions, the pathway to treatment will remain blocked regardless of the quality and availability of services. Bin Dehaish et al. (2025) reported a median delay of 17 years between hearing loss diagnosis and implantation, with the longest delays occurring prior to specialist referrals supporting the hypothesis that awareness barriers operate at the family and community level long before clinical contact is made [9]. This family-mediated pathway to care is not unique to Saudi Arabia: a qualitative study of older adults in Singapore similarly identified family members as a key cue to action, often prompting help-seeking for hearing loss before the affected individual recognized a problem themselves [11]. This convergence across culturally distinct collective societies strengthens the case that family-level awareness, rather than individual awareness alone, is a generalizable determinant of care-seeking for ARHL. Targeting this family-level awareness through culturally adapted educational campaigns represents a potentially high-impact strategy for improving early detection and management of ARHL in Saudi Arabia and across comparable collective healthcare societies worldwide.

### 4.5. Limitations

Some limitations should be considered when interpreting these findings. First, recruitment through social media, university networks, and professional channels may have skewed the sample toward more internet-active individuals, and the sample was concentrated in the Central region, limiting generalizability. Finally, as a cross-sectional online survey, causal or temporal inferences cannot be drawn, and findings should be interpreted as baseline descriptive data.

This study serves as a baseline that underscores the need for greater awareness of hearing health among adults. Future studies should include larger samples, target a wider geographic area, and explore the influence of additional factors, such as educational qualification, occupation, and prior experience with ARHL diagnosis and management, to assess their influence on awareness and perceptions.

## 5. Conclusions

This study demonstrates that adults in Saudi Arabia value good hearing and show reasonable awareness of the signs and consequences of ARHL yet harbor significant gaps in knowledge regarding its clinical course and management options. The finding that more than a third of participants believed cochlear implantation is exclusively a pediatric intervention is a notable gap in a country where this service is provided free of charge to all eligible adults. In Saudi Arabia’s collective healthcare culture, older adults typically live within extended family households and co-residing family members are the primary drivers of help-seeking behavior on their behalf. That such gaps in knowledge persist even among a comparatively young, well-educated cohort with strong access to health information is itself a telling finding, pointing to a genuine deficit in public awareness. Targeting family-level awareness through culturally adapted educational campaigns is therefore essential to bridge the gap between available services and those who need them most.

## Figures and Tables

**Table 1:** Demographic characteristics of participants.

Variable	Category	*n* (%)
**Total participants**		506 (100%)
**Gender**	Male	224 (44.3%)
	Female	282 (55.7%)
**Age group**	18–30 years	237 (46.8%)
	31–40 years	102 (20.2%)
	41–50 years	77 (15.2%)
	≥51 years	73 (14.4%)
**Region**	Central	357 (70.6%)
	Eastern	51 (10.1%)
	Western	44 (8.7%)
	Northern	24 (4.7%)
	Southern	13 (2.6%)

**Table 2:** Health-seeking behaviour of participants.

Variable	Category	*n* (%)
**Source of general medical information**	Doctor	232 (45.8%)
	Internet	180 (35.6%)
	Do not seek information	42 (8.3%)
**Source of hearing-related information**	Doctor	184 (36.4%)
	Internet	163 (32.2%)
	Do not seek information	121 (23.9%)
**Precautionary healthcare visit (no symptoms)**	No visit	199 (39.3%)
	ENT specialist	142 (28.1%)
	General practitioner	105 (20.8%)
**Preferred provider if hearing loss occurs**	ENT specialist	381 (75.3%)
	Audiologist	91 (18.0%)
	General practitioner	17 (3.4%)

**Table 3:** Responses to Question 1: To what extent do you agree with the following statements about hearing aids (HAs) and cochlear implants (CIs)?

Statement	Mean (*n*, %)
**It is usually possible to immediately identify people wearing hearing aids**	2.5 (220, 45.1%)
**Hearing implants are not externally visible**	2.8 (213, 43.6%)
**Hearing aids must be removed when the wearer goes to bed**	2.1 (306, 62.7%)
**Hearing implants are permanently fitted and so do not need to be removed before going to bed at night**	2.8 (209, 42.8%)
**Hearing aids can be a hindrance when doing sports (they slip, got lost, etc.)**	2.2 (301, 61.7%)
**Hearing implants can be a nuisance during physical activity**	2.9 (177, 36.3%)
**Hearing aids require a great deal of maintenance**	2.2 (294, 60.2%)
**Hearing implants require regular maintenance and adjustment**	2.7 (200, 41.0%)
**Qualitatively (in terms of hearing sensitivity) there is no difference between hearing aids and hearing implants**	3.6 (79, 16.2%)

Note. 1 = agree completely, 5 = do not agree at all. Mean rating shown, with *n* and percent of respondents scoring “1” or “2” in parentheses (*n* = 488 valid responses).

## Data Availability

The data presented in this study are available on request from the corresponding author. The data are not publicly available due to privacy and ethical restrictions.

## Supplementary Materials

The following supporting information can be downloaded at https://www.mdpi.com/article/10.3390/audiolres16040114/s1. Supplementary Material S1: Full questionnaire, including all 13 items and response options grouped by Health Belief Model domain.

## Abbreviations

The following abbreviations are used in this manuscript:

ARHL	Age-related hearing loss
HBM	Health Belief Model
CI	Cochlear implant
ENT	Ear, nose, and throat
GASTAT	General Authority for Statistics
SPSS	Statistical Package for the Social Sciences
IRB	Institutional Review Board
IMSIU	Imam Mohammad Ibn Saud Islamic University
KSA	Kingdom of Saudi Arabia
HA	Hearing aid
