# Supplementary material for: Age-Related Hearing Loss: Awareness, Perceptions, and Attitudes Among Adults in Saudi Arabia’s Collective Healthcare Context
Source: Audiol Res. 2026 Aug 6;16(4):114. doi: 10.3390/audiolres16040114 (PMC13509454; doi:10.3390/audiolres16040114)
Supplement: Supplementary file 1 [file audiolres-16-00114-s001.zip › audiolres-4404969-supplementary.pdf]

Supplementary Material S1

Full Questionnaire: Awareness, Perceptions, and Attitudes Toward Age-Related Hearing Loss and Cochlear Implantation

The questionnaire was adapted from the validated instrument developed by D'Haese et al. (2018), grounded in the Health Belief Model (HBM), and administered in both Arabic and English. Response options are listed beneath each question. Questions are grouped according to their corresponding HBM domain, consistent with the original instrument.

Perceived Susceptibility to Hearing Loss and Its Impact (Threat Perception)

Question 3. Which age group do you think benefits most from a hearing implant?

- Children
- Youth
- Older adults
- All age groups equally

Question 4. Which of the following statements about hearing loss are correct in your opinion?

- Hearing loss is a reduction in the ability to hear
- Hearing loss can be halted (no further deterioration)
- Hearing loss is the complete loss of the ability to hear
- Hearing loss can be cured
- Do not know / no response

(Respondents could select more than one answer.)

Question 5. Is there a difference between hearing loss and hearing impairment?

- Yes
- No
- I don't know

Question 10. What are the signs of possible hearing loss?

- Having the volume of TVs and radios turned up very high
- Difficulty hearing high-pitched sounds
- Problems and difficulties using a telephone
- Inability to locate background sounds
- Constant ringing or buzzing in the ears

(Respondents could select more than one answer.)

Question 12. What are the consequences for people suffering from hearing loss?

- Restricted communication (face-to-face and by telephone)
- Impaired quality of life
- Social isolation
- Frustration

Question 13. How important is it for you personally to have a good sense of hearing, to be able to hear well?

Rated on a 5-point scale (1 = completely agree, 5 = do not agree at all).

Perceived Barriers to Treatment

Question 1. To what extent do you agree with the following statements about hearing aids/hearing implants?

Each statement rated on a 5-point scale (1 = completely agree, 5 = do not agree at all).

- It is usually possible to immediately identify people wearing hearing aids
- Hearing implants are not externally visible
- Hearing aids must be removed when the wearer goes to bed, for example
- Hearing implants are permanently fitted and so do not need to be removed before going to bed at night
- Hearing aids can be a hindrance when doing sports (they slip, get lost, etc.)
- Hearing implants can be a nuisance during physical activity
- Hearing aids require a great deal of maintenance
- Hearing implants require regular maintenance and adjustment
- Qualitatively (in terms of hearing sensitivity) there is no difference between hearing aids and hearing implants

Question 2. Do you think there is a difference between a hearing aid and a hearing implant?

- Yes
- No
- I don't know

Current Actions Taken to Address Health Issues

Question 6. How do you find out about medical issues/illnesses?

- Doctor
- Internet
- Nothing

Question 7. How do you find out about the subject of hearing and the possible associated impairments?

- Doctor
- Internet
- Nothing

Question 8. Which of the following doctors do you visit without an acute condition, just as a precaution?

- General practitioner (GP)
- Dentist
- ENT specialist

Question 9. Why do you not visit an ear, nose, and throat (ENT) specialist as a precaution?

(Asked only of respondents who indicated in Question 8 that they do not visit an ENT specialist as a precaution.)

- Never had problems in that area
- Never thought to do so
- I do not think it's necessary

Question 11. Assuming that you suffer from hearing loss yourself, which of the following medical facilities would you visit?

- ENT specialist
- Hearing aid acoustician / audiologist
- General practitioner (GP)

Reference

D'Haese PSC, De Bodt M, Van Rompaey V, Van de Heyning P. Awareness of Hearing Loss in Older Adults: Results of a Survey Conducted in 500 Subjects Across 5 European Countries as a Basis for an Online Awareness Campaign. Inquiry. 2018;55:46958018759421. doi:10.1177/0046958018759421 PubMed PMID: 29529899.
